# Subclinical Hypothyroidism (SH) and Atherogenic Index of Plasma (AIP) in Women: A Case-Control Study From a Tertiary Care Hospital in South India

**DOI:** 10.7759/cureus.10636

**Published:** 2020-09-24

**Authors:** Madhura N.S, Mythri Shankar, Shashikala Narasimhappa

**Affiliations:** 1 Biochemistry, Institute of Nephro Urology (Government of Karnataka), Bengaluru, IND; 2 Nephrology, Institute of Nephro Urology (Government of Karnataka), Bengaluru, IND; 3 Microbiology, Institute of Nephro Urology (Government of Karnataka), Bengaluru, IND

**Keywords:** subclinical hypothyroidism, dyslipidemia, s.tsh, lipid profile, atherogenic index of plasma

## Abstract

Introduction

Subclinical hypothyroidism (SH) is defined as an increase in serum-thyroid stimulating hormone (S-TSH) above the normal level with free triiodothyronine (T4) and free thyroxine (T3) within the normal range. It is more common in females. The association between SH and dyslipidemia is unclear. There are many controversial studies regarding the same. This is the single largest study of Atherogenic index of plasma (AIP) in SH among women from our country. Our aim is to study the lipid profile and AIP in SH patients. We will also study the correlation between AIP and S-TSH levels in SH patients.

Materials and methods

It was a retrospective study conducted in a tertiary care center. A total of 97 females with SH were taken as cases and 86 normal females were taken as euthyroid (ET) controls. They were matched for demographic characteristics. T3, T4, S-TSH, total cholesterol, S-triglycerides (S-TG), high-density lipoprotein - cholesterol (HDL-C), low-density lipoprotein cholesterol (LDL-C), and AIP were compared between the two groups. Spearman’s correlation between TSH and AIP was studied in the SH group. Mann-Whitney U test was performed.

Results

The TG, HDL, AIP levels were significantly different between both groups. TG, AIP was higher in the SH group compared to the ET group (p value of TG= 0.01, p value of AIP <0.0001). HDL was lower in the ET group compared to the SH group (p value <0.0001). AIP showed a significant positive correlation with S-TSH levels in the SH group. (r value=0.72, p value=<0.001).

Conclusion

It is important to regularly monitor SH patients for dyslipidemia, in order to start early therapy with levothyroxine/statins. Emphasis should be laid on lifestyle changes such as diet and exercise from the time of diagnosis. Community level education and awareness should be encouraged. Also, AIP is a better parameter to assess cardiovascular risk in SH patients than a conventional lipid profile.

## Introduction

Subclinical hypothyroidism (SH) is a state of isolated elevation of thyroid-stimulating hormone (TSH) levels with a normal level of free triiodothyronine (T4) and free thyroxine (T3) hormones. Worldwide, it is more common in females with a prevalence of 7.5%-8.5%, whereas it is 2.8%-4.4% in males [1,2].

It has been proved by many studies that overt hypothyroidism is associated with dyslipidemia and hence increased cardiovascular risk [3,4]. However, studies related to SH and cardiovascular risk have shown conflicting results [5]. One study has also indicated that a minor change in TSH within the normal range can also predispose to increased cardiovascular risk [6].

At present, serum lipid ratios like triglyceride/high-density lipoprotein cholesterol (TG/HDL-C) and low-density lipoprotein cholesterol/high-density lipoprotein cholesterol (LDL-C/HDL-C) predicts cardiovascular risk better than conventional lipid profiles [7]. Small dense lipoprotein (sdLDL) is a predominant risk factor for cardiovascular disease and AIP is a good surrogate marker for sdLDL [7]. Relationship between thyroid hormones and lipid profiles is very complex and there are only a few studies in this context.

AIP is a new index. It is a logarithmic ratio of TGs and HDL-C [8]. It is a marker of dyslipidemia and diseases associated with it such as cardiovascular disease and cerebrovascular disease [9-11]. 

We are all aware of lipid abnormalities associated with overt hypothyroidism, which has been established by many studies. However, the association between SH and lipid abnormalities is not clearly established and requires further research. There is also a paucity of data in the Indian population, especially the female gender. Also, there is no study based on the correlation between SH and AIP exclusively in women from our country. Our study is the only largest study in women from our country till date. Hence, our aim is to study the association between SH and lipid profile in women. We also intend to study the correlation between SH and AIP in this study.

## Materials and methods

This was a retrospective case-control study conducted at a South Indian tertiary care center for Nephrology and Urology services. A detailed analysis of the medical case records was done. Data was collected from the laboratory records of patients over a period of nine months from January 2019 to September 2019. Institutional Ethical Committee clearance was obtained - [IE - R 456/2].

Inclusion criteria

Adult women between the age group of 20 to 60 years who visited the Outpatient department were included in the study. Thyroid profile values (TSH, T3, T4) were recorded.

Cases: Women with TSH between 5.0 - 10μIU/mL and with normal free T3 and free T4 were defined as cases and were recruited under the SH group.

Controls: Women with normal thyroid levels were taken as controls and were recruited under the euthyroid (ET) group. A total of 97 cases in the SH group and 86 in the ET group were included.

Exclusion criteria

Women with other diseases like renal insufficiency, hepatic disease, overt hypothyroidism, diabetes mellitus, and were excluded from the study.

Lipid profile parameters including total cholesterol, TGs, LDL-C and HDL-C were assessed in these patients. Atherogenic index of plasma (AIP) was calculated using the formula AIP=log (TG/HDL) where TG is triglycerides and HDL is high-density lipoprotein. Many studies have shown that AIP predicts cardiovascular risk. Values of AIP <0.1 are considered low risk, 0.1 - 0.2 is intermediate risk and, more than 0.2 is at high risk for cardiovascular diseases [8].

All the lipid profile parameters and thyroid profile parameters were measured in the biochemistry laboratory using the Abbott analyzer ci4100 (Abbott Laboratories, Chicago, Illinois, USA) by electro-chemiluminescence immunoassay (ECLIA) with the serum sample collected using all aseptic precautions.

Statistics

Statistical Package for the Social Sciences (SPSS) version 23 (SPSS Inc., Chicago, IL) was used to do statistical analysis. The mean and standard deviation was used for continuous variables. Mann Whitney U test was used to compare thyroid profile, lipid profile, and AIP between both the groups. P value of less than 0.05 was considered significant. Spearman's correlation was used to correlate between AIP and TSH values in the SH group.

## Results

Clinical and statistical data of the two groups (SH group and ET group) are summarized in Table 1.

**Figure 1:**
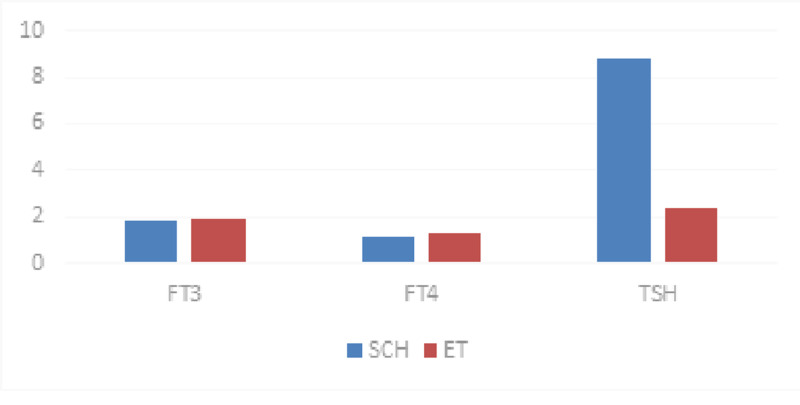
Comparison of thyroid profile between the SCH group and the ET group SCH: subclinical hypothyroid, ET: euthyroid.

Age was matched between the two groups. Only females were included in the study. Free T4 (p=0.008) and TSH (p<0.001) was significantly increased in the SH group compared to the ET group. However, free T3 (p=0.4) showed no significant difference between the two groups (Table 1) (Figure 1).

**Table 1:** Clinical and statistical data of the study groups p value <0.05 is significant FT3: free thyroxine, FT4: free triiodothyronine, TSH: thyroid-stimulating hormone, TC: total cholesterol, LDL: low-density lipoprotein, HDL: high-density lipoprotein, AIP- atherogenic index of plasma, Ref- reference range.

Parameters	SH group(n=97) MEAN ±SD	ET group(n=86) MEAN ±SD	P VALUE	Significance level
Age	46±10	47±7	0.43	Not significant
FT3 Ref:(1.71-3.71pg/ml)	1.83±0.6	1.91±0.7	0.40	Not significant
FT4 Ref:(0.7-1.48 ng/dl)	1.1±0.3	1.3±0.1	0.008	significant
TSH Ref: (0.5-4.9 mIU/ml)	8.8±1.6	2.4±1.6	<0.001	significant
TC Ref:(0-200 mg/dl)	185±59	174±28	0.104	Not significant
TG Ref:(0-150 mg/dl)	182±77	162±16	0.01	significant
HDL Ref: (40-60 mg/dl)	32±12	48±7	<0.0001	significant
LDL Ref: (0-100 mg/dl)	109±46	100±32	0.124	Not significant
AIP (log TG/HDL)	0.395±0.01	0.111±0.01	<0.0001	Significant

**Figure 2:**
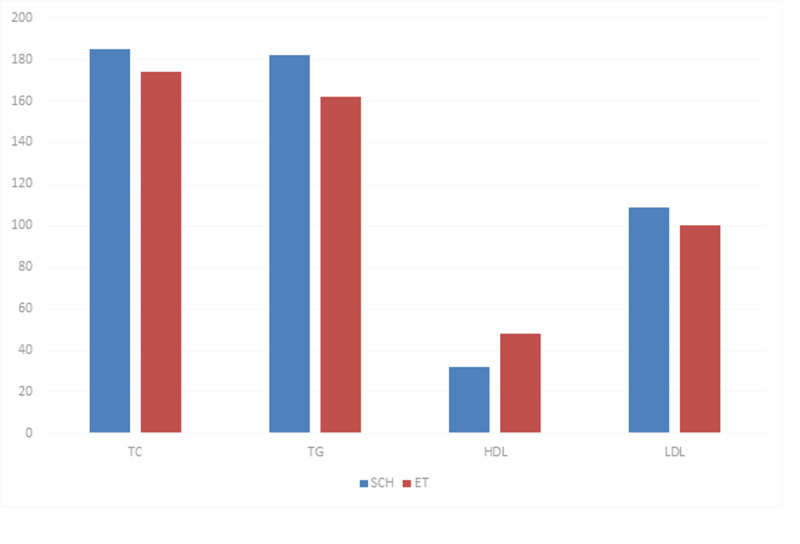
Comparision of lipid profile between the SCH group and ET group SCH: subclinical hypothyroid, ET: euthyroid, TC: total cholesterol, TG: triglycerides, HDL: high-density lipoprotein, LDL: low-density lipoprotein.

On comparison of the lipid profile markers, triglycerides (p=0.01) and HDL cholesterol (p<0.0001) were significantly increased in the SH group compared to the ET group while total cholesterol and LDL were not significantly different between the two groups (Figure 2).

**Figure 3:**
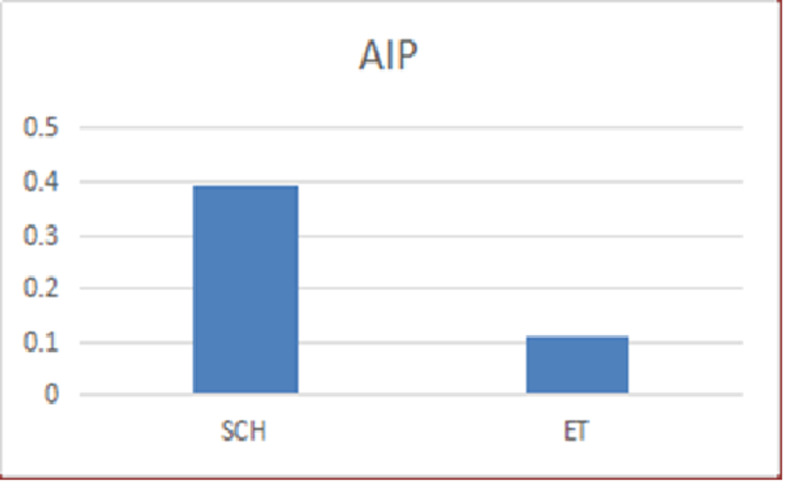
Comparison of AIP between between the SCH group and ET group AIP: atherogenic index of plasma, SCH: subclinical hypothyroid, ET: euthyroid.

AIP was significantly higher in the SH group, compared to the ET group (p<0.0001), and the mean value of AIP in SH group was 0.395 which indicates high risk for cardiovascular disease in these patients (Figure 3).

**Figure 4:**
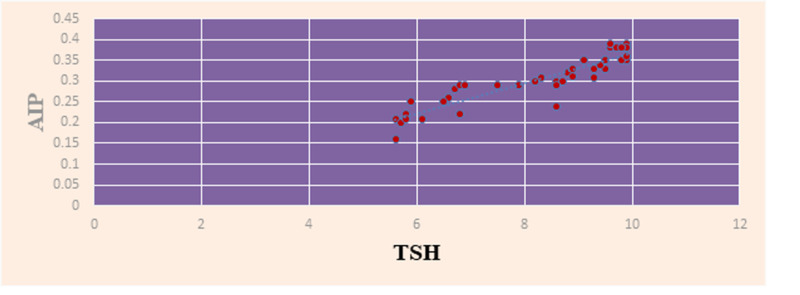
Scatter diagram showing correlation between TSH and AIP in subclinical hypothyroid patients TSH: thyroid stimulating hormone, AIP: atherogenic index of plasma.

The correlation between TSH level and AIP was assessed in SH patients using the Pearson correlation test. There was significant positive correlation (r=0.72) between AIP and TSH in the SH patients (p<0.001) (Figure 4).

## Discussion

Primary hypothyroidism is defined as a decreased thyroid activity which results from reduced secretion of both T4 and T3 with elevated TSH levels due to its hypersecretion from the pituitary as a result of feedback mechanism activating the hypothalamo-pitutary axis [12]. Though symptoms of hypothyroidism are nonspecific, clinical suspicion of hypothyroidism can be made with the presence of multiple symptoms of slowing of body functions of recent onset [13,14].

Unlike hypothyroidism, SH is purely laboratory diagnosis, as these patients will not present with clinical manifestations, but they might progress to overt hypothyroidism in 2%-5% cases every year [15].

The association between SH and dyslipidemia has been studied in various countries. However, the association of SH with the lipid ratios and AIP has been rarely studied [2,6].

Hence, we have retrospectively conducted a case-control study where we have included all patients with SH as cases and compared them with normal controls. We have compared all parameters of lipid profile, lipid ratios, and AIP.

A study by Vanderpump et al. in 2002 [16], on the epidemiology of hypothyroidism and SH, have proved that the prevalence of overt hypothyroidism is ten times more common in women compared to men, while SH was prevalent in 8% of women and 3% of men. Following this, many studies all over the world have indicated the higher prevalence of hypothyroidism and SH in women than in men. Hence in the present study, we have included only the females between 20-60 years.

In our study, age was matched between the two groups. The mean age in the SH group was 46 years and in the ET group was 47 years. There was no significant difference between the two groups. TSH was significantly higher in the SH group compared to the ET group. This is in concordance with the study by Karthick et al. in Tamil Nadu [6]. The mean T4 was lower in the SH group compared to the ET group. However, the values were within the normal reference range. This difference was significant (P value=0.008). There was no significant difference seen in the values of T3 between both the groups (P value=0.4). A study by Karthick et al. [6] showed no significant difference in the values of T3 and T4 between both the groups. Another study by James et al. [17] showed a significant difference in both T3 and T4 values between the SH group and the ET group. This difference in results may be due to the higher sample size in our study. We had a sample size of 97 in the SH group, whereas the studies by Karthick et al. [6] and James et al. [17] had a lower sample size of 30 and 70, respectively.

With respect to lipid profile parameters, in our study, TG was significantly higher in the SH group compared to the ET group. A study by James et al. [17] also demonstrated similar results. Many other studies conducted in India and abroad [2,4] have shown an increase in TG in the SH group, however, they have also demonstrated an increase in TC and LDL in the SH group. Our study did not show any significant difference in LDL and TC between both the groups.

Also, in our study, HDL was significantly lower in the SH group compared to the ET group. This is in concordance with the study by Karthick et al. [6]. As opposed to this, James et al. [17] did not show any significant difference in the values of HDL between both the groups. The lower HDL levels in our study can be attributed to the sedentary lifestyle of our population. Many studies have suggested an improved lipid profile parameter following treatment with thyroxine. However, we have not studied the treatment aspect [18].

In our study, AIP was significantly higher in the SH group compared to the ET group. The mean AIP in the SH group was 0.39 and in the ET group, it was 0.11. The higher values in the SH group fall in the at-risk category ( >0.11) for cardiovascular diseases. AIP has already proven to be closely associated with atherogenic fractions of LDL lipoprotein which is sdLDL [7]. AIP is more predictive of cardiovascular disease compared to individual lipids [19].

We performed an extensive literature search. This is the only study in India to compare the AIP and SH with a maximum sample size, till date. Also, ours is the only study to focus entirely on women. However, there are studies of AIP in obesity, metabolic syndrome, and diabetes mellitus population. In these studies, AIP has proven to be a good marker for cardiovascular risk [7,20].

We have also studied the association between TSH levels and AIP. There was a significant positive correlation (r value= 0.72). The values of AIP increased with increasing TSH levels. Hence, patients with higher TSH levels were at higher cardiovascular risk. One study conducted in India has shown an increase in dyslipidemia with overt hypothyroidism (TSH >10mIU/L). There was no dyslipidemia seen in patients with TSH < 10mIU/L [21].

This further proves that more studies are required to study the association between AIP and SH.

Summarizing our study, we have included only those with TSH between 5 to 10 mIU/L in the SH group considering the fact that those with TSH more than 10 mIU/L might present with clinical symptoms of hypothyroidism. In our study, all lipid markers, TC, TG, LDL were high in SH compared to normal controls. But this difference was significant for TG and not significant for TC and LDL. HDL was significantly low in SH than in normal controls.

The limitation of the study was that it was a retrospective study, based on hospital records. More prospective studies are required to study dyslipidemia in SH patients in depth. The molecular basis of SH and dyslipidemia can only be studied in animal models.

Hence, it is important to counsel the patients with SH about diet and lifestyle modifications in order to prevent dyslipidemia at a community level. All patients with SH should be monitored for dyslipidemia on a regular basis. Even if the lipid profile is normal, AIP may be abnormal. Hence it is important to calculate AIP as well to assess cardiovascular risk. Regular monitoring will help in the early treatment of the disease with levothyroxine/statins and the prevention of morbidity and mortality.

## Conclusions

As there are no studies in India till date with such a large sample size, our study could be a trend setter for future research in the field of SH and dyslipidaemia. Also, there are hardly any studies on AIP assessment in SH patients. Research in AIP holds great potential. Our study proves that SH is associated with dyslipidemia and a high AIP, suggestive of higher cardiovascular and cerebrovascular disease risk. This calls for strict monitoring of SH patients, early detection of dyslipidemia, and early initiation of treatment.
